# The Transcriptional Effects of PCB118 and PCB153 on the Liver, Adipose Tissue, Muscle and Colon of Mice: Highlighting of Glut4 and Lipin1 as Main Target Genes for PCB Induced Metabolic Disorders

**DOI:** 10.1371/journal.pone.0128847

**Published:** 2015-06-18

**Authors:** Aurélia Mesnier, Serge Champion, Laurence Louis, Christophe Sauzet, Phealay May, Henri Portugal, Karim Benbrahim, Joelle Abraldes, Marie-Christine Alessi, Marie-Josephe Amiot-Carlin, Franck Peiretti, Philippe Piccerelle, Gilles Nalbone, Pierre-Henri Villard

**Affiliations:** 1 IMBE (Institut Méditerranéen de Biodiversité et d'Ecologie Marine et Continentale), UMR CNRS 7263, IRD 237 Aix-Marseille Université Avignon Université, Campus Timone, Faculté de Pharmacie, 27 Boulevard Jean Moulin, F-13385, Marseille cedex 05, France; 2 UMR S 910 Génétique Médicale et Génomique Fonctionnelle, Campus Timone, Faculté de Médecine, 27 Boulevard Jean Moulin, F-13385, Marseille cedex 05, France; 3 UMR INSERM 1062, INRA 1260, Nutrition, Obésité et Risque Thrombotique (NORT), Aix-Marseille Université Campus Timone, Faculté de Médecine, 27 Boulevard Jean Moulin, F-13385, Marseille cedex 05, France; Tohoku University, JAPAN

## Abstract

Epidemiological studies have associated environmental exposure to polychlorinated biphenyls (PCBs) with an increased risk of type 2 diabetes; however, little is known about the underlying mechanisms involved in the metabolic side-effects of PCB. Our study evaluated the transcriptional effects of a subchronic exposure (gavage at Day 0 and Day 15 with 10 or 100 μmol/Kg bw) to PCB118 (dioxin-like PCB), PCB153 (non-dioxin-like PCB), or an equimolar mixture of PCB118 and PCB153 on various tissues (liver, visceral adipose tissue, muscle, and colon) in mice. Our results showed that a short-term exposure to PCB118 and/or PCB153 enhanced circulating triglyceride levels but did not affect glycemia. Among the studied tissues, we did not observe any modification of the expression of inflammation-related genes, such as cytokines or chemokines. The main transcriptional effects were observed in visceral adipose and liver tissues. We found a downregulation of lipin1 and glut4 expression in these two target organs. In adipose tissue, we also showed a downregulation of Agpat2, Slc25a1, and Fasn. All of these genes are involved in lipid metabolism and insulin resistance. In muscles, we observed an induction of CnR1 and Foxo3 expression, which may be partly involved in PCB metabolic effects. In summary, our results suggest that lipin1 and glut4, notably in adipose tissue, are the main targeted genes in PCB-induced metabolic disorders, however, further studies are required to fully elucidate the mechanisms involved.

## Introduction

Polychlorinated biphenyls (PCBs) are a type of organochlorine and are persistent organic pollutants (POPs). PCBs are very stable and resistant to temperature and pressure extremes; in addition, these compounds are fat-soluble, which leads to bioaccumulation in individuals and the food chain.

PCBs have been widely used for industrial purpose over the 50 years, they were banned since 1979 in the U.S. and other developed countries. There are 209 possible PCB congeners that are categorized based on structural properties, such as the number of chlorine atoms in the compound. A common classification divides PCBs into dioxin-like (which are agonists of the Ah receptor (AhR)) and non-dioxin-like (which are agonists of constitutive androstane receptor (CAR) or pregnane X receptor (PXR)) based on their structural and toxicological similarity with the dioxin molecule.

Human exposure occurs mainly from consumption of PCB-containing foods and inhalation of contaminated air. Fish (especially when taken from lakes or rivers with high levels of PCBs), meat and dairy products are the main dietary sources of PCBs.

Human exposure to PCBs is associated with cancers, including non-Hodgkin lymphoma; furthermore, PCBs have been classified as probable carcinogens to humans [1]. PCB bioaccumulation can lead to reduced infection fighting ability, increased rates of autoimmunity, cognitive and behavioral problems, and hypothyroidism [2]. Some research also links PCBs to increased rates of type 2 diabetes from decreased insulin sensitivity in peripheral tissues. The relationship of PCBs with diabetes has been investigated in several cross-sectional studies and longitudinal studies. The evidence from cross-sectional studies supports an association of dioxin-like PCBs with diabetes (diagnosed plus undiagnosed diabetes). The association of non-dioxin-like PCBs and diabetes has been reported; however, this association was not significant when PCBs were examined with other POPs. Few longitudinal studies of PCBs and diabetes have been completed. One study concluded that only women show an association between PCBs and diabetes; however, three studies found no association for elevated PCBs and diabetes (for review see [3]). Despite the extensive research about relationships between PCB exposure and diabetes, the underlying mechanism is not well understood. One potential mechanism involves the regulation and metabolism of glucose and insulin. Olsen et al. showed that TCDD, PCB77, and PCB52 reduced functional Glut4 (glucose transporter protein) in preadipocyte cells lines through AhR binding [4]. Ruzzin et al. [5] studied the development of insulin resistance in adult male rats exposed to crude salmon oil containing persistent organic pollutants. These authors proposed that POPs affect nuclear receptors (AhR, CAR, and pregnane X receptor), which increase chronic low grade inflammation, decrease mitochondrial function and fatty acid oxidation, and increase lipogenesis to ultimately produce insulin resistance syndrome. In rat liver studies, TCDD affected the mRNA expression of numerous genes related to glucose and insulin sensitivity, including decreases in glucokinase and fructose-6-phosphatase transport protein 1 [6]. Other studies have focused on the peroxisome proliferator-activated receptor (PPAR) and AhR. PPAR plays a role in glucose homeostasis and the translation of Glut4. Dioxins, such as TCDD, bind to the AhR and antagonize PPAR and its associated functions; thus, the alteration of this pathway may be another explanation for the link between PCBs and diabetes [7]. Further studies are needed to fully elucidate the underlying genetic and biochemical processes involved in PCB-induced type 2 diabetes.

Our study evaluated the genomic effects of a subchronic exposure to PCB118 (dioxin-like PCB), PCB153 (non-dioxin-like PCB), or an equimolar mixture of PCB118 and PCB153 in various mouse tissues (liver, visceral adipose tissue, muscle, and colon). We have chosen these two PCBs because humans are mainly exposed to PCBs through the consumption of PCB-containing fishes, and PCB118 and PCB153 are frequently found in wild fishes [8]. Among the studied tissue, liver, visceral adipose tissue, and muscle are well defined target tissues for type 2 diabetes. But, colon is of interest, since glucagon-like peptide 1 (GLP-1) is a gut-derived hormone that enhances glucose-induced insulin secretion. In addition to its pancreatic effects, GLP-1 can induce metabolic actions by interacting with its receptors expressed on nerve cells in the gut and the brain [9].

## Materials and Methods

### Chemicals

PCB118 and PCB153 were purchased from AccuStandard Inc. (New Haven, USA).

### Animal treatments and sample collection

All experiments were approved by the Animal Protection Committee of the Mediterranean University (approval N°20120102). Male C57BL/6 mice (2 months of age; Charles River Laboratories, Les Oncins, France) were housed in a pathogen-free environment and given access to food (Diet for maintenance A04, from “Safe”, Augy, France) and water *ad libitum*. Daily, mice were observed in order to detect any suffering.

To minimize the contamination from pesticide residues, PCBs were dissolved in olive oil produced from organic farming. Treatments were administered by gavage at Day 0 and Day 15.

Mice were randomly divided into 7 groups of 10 animals and given the following treatments: control (olive oil), PCB118 10 μmol/Kg bw, PCB118 100 μmol/Kg bw, PCB153 10 μmol/Kg bw, PCB153 100 μmol/Kg bw, an equimolar mixture of PCB118 and PCB153 10 μmol/Kg bw, or an equimolar mixture of PCB118 and PCB153 100 μmol/Kg bw. The highest doses of PCB studied in our study, are comparable to those of precedent published studies performed in mice [10–11]. After treatment, no suffering of mice was observed.

At Day 29, mice were fasted. At Day 30, mice were euthanized by the intraperitoneally injection of a lethal dose of thiopental (150 mg/Kg bw), blood samples were collected retro-orbitally, and after cervical disruption, tissues (liver, visceral adipose tissue, muscles, and colon) were collected. After blood centrifugation, plasma was collected for biochemical analyses. The liver and colon samples were quickly frozen in liquid nitrogen and stored at -80°C until further use. Visceral adipose tissue and muscles were preserved in Allprotect Tissue Reagent (Qiagen, Courtaboeuf, France) at 4°C until further use.

### Biochemical analyses

Biochemical analyses were performed on a Modular PP (Roche Diagnostics, Meylan, France) at the Clinical Laboratory of the Hospital "La Conception" (Marseille, France). We determined the levels of glucose, total cholesterol, HDL cholesterol, LDL cholesterol, triglycerides, phospholipids, alkaline phosphatase, alanine aminotransferase (ALT), and aspartate aminotransferase (AST).

### RNA extraction

Liver, muscle, and colon RNA were extracted using a Qiagen RNeasy kit (Qiagen, France). Visceral adipose tissue RNA was extracted using Qiazol reagent (Qiagen, France) and then further purified on a column from the RNeasy kit.

The concentration of RNA samples was assessed using a NanoDrop Lite (Fisher Scientific France, Illkirch, France). The quality of RNA samples was confirmed using a Bioanalyzer Agilent 2100 (Agilent Technologies, Courtaboeuf, France).

For gene expression microarrays and real-time RT-PCR experiments, RNA samples of 3 mice from each treatment group were randomly pooled and analyzed.

### Gene expression microarray and microarray data analysis

After assessing the RNA quality, liver, muscle, colon and adipose tissue samples with a RNA Integrity Number (RIN) greater than 7.0 were selected for further analysis (PCB, controls and concentrations). Samples were labeled with Agilent two-color Quick Amp labeling kits using a total RNA input ranging from 600 to 1000 ng. Labeled cRNA was analyzed using the Nanodrop to ensure that the amplification and labeling reactions were sufficient. Labeled cRNA was hybridized on 18 Agilent 4x44K microarrays. Samples were then scanned on an Agilent DNA scanner. Raw data were extracted using Feature Extraction software and analyzed with GeneSpring. Lastly, raw data from the 3 sample pools for each tissue (liver, colon, muscle and adipose tissue) were processed for statistical analysis (Student’s t-test, P < 0.05). Only changes greater than 2-fold were taken into account for data analyses.

### Real-time RT-PCR

Reverse transcription was completed using 1 μg of pooled RNA and a High capacity cDNA reverse transcription kit as specified by the manufacturer (Applied Biosystems, France).

PCR experiments were performed using a LightCycler480 System (Roche, France) in 384 well-plates using a liquid handler automated workstation (Biomek 3000 Beckman). The PCR was performed with 0.4 μM of each primer and EXPRESS SYBR GreenER qPCR Supermix (Invitrogen, France). Cycling conditions including a 2 min UDG activation at 50°C, 2 min activation at 95°C followed by 40 cycles of amplification (15 s denaturation at 95°C, 1 min primer annealing and fragment elongation at 60°C). A melt program was performed at the end of the PCR. The raw fluorescence data were analyzed using LightCycler480 software. Target gene mRNA expression was normalized to β-actin, and data were quantified using the 2־^∆∆Ct^ method. The primers used are listed in Table 1.

**Table 1:** Primers used for real-time qRT-PCR experiments.

Primer	Sequence (5'-3')	Primer	Sequence (5'-3')
Mus_ActB_F	GGAGGGGGTTGAGGTGTT	Mus_Lipin1_F	TCCGGAAGTTAAATTATTTTCTTTG
Mus_ActB_R	GTGTGCACTTTTATTGGTCTCAA	Mus_Lipin1_R	TGACGGCTATAATGCCTAAGGA
Mus_CD28_F	AGCTGTTTTGGGCACTGGT	Mus_Lipin2_F	AACAATGTAGAGTGGACCAGGAC
Mus_CD28_R	GAGCCACTGTCACTAGCAAGC	Mus_Lipin2_R	AGGCTGCAGGGAACAGAG
Mus_Foxo3_F	AGCAACATGGGCTTGAGTG	Mus_Slc25a1_F	GCCATGGTCAGGTCACAGT
Mus_Foxo3_R	GAGACTGCTGCTGGTGTTTG	Mus_Slc25a1_R	GAGGCAGCTGTGGTAGGC
Mus_Serpin1_F	TTCCAGGGGTGTGATTGAATA	Mus_Pfkfb3_F	CAGAGGACAGCTTGACAGGA
Mus_Serpin1_R	CCGTATGCCACGGTTTAAATA	Mus_Pfkfb3_R	GAGGCCTGGGCTCAATCT
Mus_IL2_F	GCTCTACAGCGGAAGCACA	Mus_Enolase1_F	GAGGCGCTTAGTGCTGCT
Mus_IL2_R	GAGCTCCTGTAGGTCCATCAA	Mus_Enolase1_R	CTGCAAAGCAAGGAAAGAGG
Mus_IL6_F	ACGGCCTTCCCTACTTCAC	Mus_Pygb_F	CGGCTGAAGCAGGAGTACTTT
Mus_IL6_R	ACAGGTCTGTTGGGAGTGGT	Mus_Pygb_R	TTGAACCTTCGAATGATGTCC
Mus_IL1BETA_F	CAAAAGATGAAGGGCTGCTT	Mus_Pnliprp1_F	TGCCAACAATGTGCGAGTAG
Mus_IL1BETA_R	GAAGCTGGATGCTCTCATCA	Mus_Pnliprp1_R	CTCACAAGGATGTCAATCATCTG
Mus_Gck_F	CTGGATGACAGAGCCAGGAT	Mus_Pla2g1b_F	CACCGGGAAATTCTGTTAGC
Mus_Gck_R	GCTGGAACTCTGCCAGGAT	Mus_Pla2g1b_R	GGGTGAAATAAGACAGCAAGGT
Mus_Slc2a4_F	TCACTGTTTGAAGATGAGTGTCC	Mus_Agpat2_F	TCACCTCAGGAACAATCAAGG
Mus_Slc2a4_R	GGGTGAGTGAGGCATTTTCT	Mus_Agpat2_R	TCTGTCAGACCATTGGTAGGG
Mus_Cyp1a1_F	TCTTTTGGGAGGAAGTGGAA	Mus_Lipc_F	CAAGGCGTGGGAACAGAG
Mus_Cyp1a1_R	TCCATACATGGAAGGCATGA	Mus_Lipc_R	TGGCTTCTTTAATGGCTTGC
Mus_Cyp1a2_F	CTGGCTTTGACACAGTCACC	Mus_Fasn_F	GTGGGAGGACAGAGATGAGG
Mus_Cyp1a2_R	GGCCATGTCACAAGTAGCAA	Mus_Fasn_R	GCTGGAGCACAAGGAACG
Mus_Cyp2b9_F	CCCAAAGAGAGTGGTATTGGA	Mus_Sult1e1_F	TCCCAGAATAGTAAAAACTCACCTG
Mus_Cyp2b9_R	CCAATTAGCGGGCTAAGAAGT	Mus_Sult1e1_R	TGCAATTCTTTTCCCAAAATG
Mus_Cyp2c54_F	TTTATCAAGAGTTTATGGTCCTGTGTA	Mus_Ifng_F	GCAAAAGGATGGTGACATGA
Mus_Cyp2c54_R	CATATCCATGCAACACCACAG	Mus_Ifng_R	TTCAAGACTTCAAAGAGTCTGAGGTA
Mus_Cyp2c70_F	CCACAGTGAAATATGGGCTTTT	Mus_Itgb6_F	CCTGGCCTTGAACTCACAAC
Mus_Cyp2c70_R	CTGAATTTTAGCTGTGACTTCTGG	Mus_Itgb6_R	CTCTGAGCATGAAATGGAAGG
Mus_Gpx3_F	ACAATTGTCCCAGTGTGTGC	Mus_Tank_F	GACATAGTCTGCGAAGGAACG
Mus_Gpx3_R	ACCATCCCTGGGTTTCAAG	Mus_Tank_R	TTGAGTTGCTCACCAATGTTTT
Mus_Gsta1_F	CTTCTGACCCCTTTCCCTCT	Mus_Alox5_F	CACTGTTCTCTTTCGATCAACAAT
Mus_Gsta1_R	GCTGCCAGGCTGTAGGAAC	Mus_Alox5_R	GGGGCAAAGACCTTGTCA
Mus_Gsta2_F	CAAATTGAAGAAGCAAGGAAGG	Mus_Mgat2_F	CTGGTGTACCAGTTGAACTTCG
Mus_Gsta2_R	GCCAGTATCTGTGGCTCCAT	Mus_Mgat2_R	CCAGGTGCCGTCATTACC
Mus_Gsto2_F	CTGCTGCAGTGGAAAGTGAG	Mus_Pde8b_F	CGAGTCTCCAACTTTGTTTCG
Mus_Gsto2_R	ACAGTCGACAGCACTCTTGC	Mus_Pde8b_R	TGTTTTTGTTTCTGAGGTCCTG
Mus_Pla2g1b_F	CACCGGGAAATTCTGTTAGC	Mus_Cnr1_F	GCACCTTCACGGTTCTGG
Mus_Pla2g1b_R	GGGTGAAATAAGACAGCAAGGT	Mus_Cnr1_R	GACTGCGGGAGTGAAGGAT
Mus_Smpd3_F	AGAGCAGGGCTGACTCCA	Mus_Rsg2_F	ACGAAAACCCCAAGTTTCCT
Mus_Smpd3_R	TGGCTCTAGTCACACGTTGG	Mus_Rsg2_R	GAGTAGCACAGATTATTAGCCAAATG
Mus_Gyk_F	AGGCCTAGGAGAATGCAGGT		
Mus_Gyk_R	CCTAGGCAGCCCCTTTACTC		

### Statistical analysis

A statistical analysis was performed with an ANOVA followed by a Dunnett’s test using GraphPad Prism (GraphPad Software). Values were considered significantly different at P < 0.05. The results are presented as the means ± SD.

## Results

### Effect of PCB treatment on weight and biochemical parameters

Changes in body weight in the different groups are summarized in Table 2. We did not observe any significant variation in weight among the different treatments, except a slight decrease with the low dose of PCB118 at Day 29.

**Table 2:** Changes in body weight in the different groups (*p < 0.05).

	Control	PCB11810 μmol/kg	PCB118100 μmol/kg	PCB15310 μmol/kg	PCB153100 μmol/kg	PCB Mix10 μmol/kg	PCB mix 100 μmol/kg
**D0**	24.10 ± 1.48	22.90 ± 0.74	23.90 ± 0.94	23.20 ± 1.20	22.90 ± 0.92	24.00 ± 1.00	23.30 ± 1.36
**D7**	25.17 ± 1.67	23.75 ± 0.83	24.86 ± 0.78	23.93 ± 1.22	23.91 ± 1.15	25.08 ± 1.09	24.12 ± 1.12
**D15**	26.35 ± 1.81	24.62 ± 0.89	25.96 ± 0.66	24.99 ± 1.35	24.97 ± 1.03	26.26 ± 1.17	25.21 ± 1.31
**D21**	26.83 ± 1.73	25.41 ± 1.01	26.69 ± 0.93	25.62 ± 1.23	25.77 ± 1.09	26.97 ± 1.05	25.84 ± 1.07
**D29**	28.27 ± 1.48	25.43 ± 0.76*	27.40 ± 0.67	26.25 ± 1.72	26.83 ± 1.27	27.88 ± 0.54	25.64 ± 1.11

D0: start of the treatment. D7: 8th day of treatment. D15: 16th day of treatment. D21: 22h day of treatment. D29: 30th day of treatment.

The results of the biochemical analyses are summarized in Table 3. PCB exposure did not induce hepatic injury at the doses used. The circulating levels of alkaline phosphatase, ALT and AST in all treated groups were similar to the control group. We did not observe any changes in the circulating levels of glucose, cholesterol (total, HDL and LDL), and phospholipids. Triglyceride levels were significantly enhanced in PCB-exposed mice, and the highest effects were observed with low doses of PCB118 and PCB153 (1.8-fold and 1.6-fold, respectively).

**Table 3:** Biochemical parameters from the different groups (*p < 0.05).

	Control	PCB118 10 μmol/kg	PCB118 100 μmol/kg	PCB153 10 μmol/kg	PCB153 100 μmol/kg	PCB mix 10 μmol/kg	PCB mix 100 μmol/kg
Glucose (mmol/L)	8.6 ± 2.3	7.6 ± 1.3	7.1 ± 0.8	7.3 ± 1.0	6.6 ± 0.6	6.6 ± 0.6	6.5 ± 1.0
Total Cholesterol (mmol/L)	2.1 ± 0.1	2.2 ± 0.1	2.1 ± 0.1	2.2 ± 0.2	1.9 ± 0.2	2.2 ± 0.1	2.1 ± 0.4
HDL Cholesterol (mmol/L)	2.0 ± 0.1	2.0 ± 0.1	1.9 ± 0.1	1.9 ± 0.3	1.8 ± 0.2	2.0 ± 0.2	1.9 ± 0.3
LDL Cholesterol (mmol/L)	0.2 ± 0.0	0.1 ± 0.0	0.1 ± 0.0	0.1 ± 0.1	0.1 ± 0.0	0.2 ± 0.3	0.2 ± 0.0
Triglycerides (mmol/L)	0.5 ± 0.1	0.9 ± 0.1*	0.7 ± 0.1*	0.8 ± 0.2*	0.5 ± 0.1	0.6 ± 0.1	0.6 ± 0.1
Phospholipids (mmol/L)	2.1 ± 0.1	2.4 ± 0.2	2.3 ± 0.1	2.4 ± 0.2	2.0 ± 0.3	2.4 ± 0.2	2.2 ± 0.2
Alkaline Phosphatases (UI/L)	66.6 ± 9.3	75.8 ± 5.6	69.5 ± 10.3	73.9 ± 13.0	75.9 ± 8.0	73.4 ± 4.5	67.4 ± 18.8
A.S.T. (UI/L)	47.7 ± 9.2	40.9 ± 4.0	57.6 ± 25.3	45.8 ± 12.2	55.6 ± 11.0	48.9 ± 11.8	48.7 ± 12.2
A.L.T. (UI/L)	16.1 ± 2.7	15.3 ± 2.8	18.8 ± 6.2	15.3 ± 4.0	15.0 ± 3.5	14.3 ± 2.8	16.0 ± 6.0

### Results of microarray analyses

The expression of approximately 1,000 genes was significantly modulated by PCB exposure. We selected genes that showed a minimum of a 2-fold (up or down) change in expression in at least two treated groups for further analyses.

The results of gene expression changes within multiple pathways are summarized in Tables 4–7.

**Table 4:** Genes involved in the metabolism of xenobiotics based on microarray analyses.

**LIVER**						
**Gene**	**PCB118-10**	**PCB118-100**	**PCB153-10**	**PCB153-100**	**PCB MIX-10**	**PCB MIX-100**
Cyp1a1		211				59.7
Cyp1a2		4.8				3.9
Cyp2b9		12.6	9.4	30.6	2.5	26.3
Cyp2b10		14.0	9.5	33.9	2.6	28.7
Cyp2c54				2.5		2.0
Cyp2c70	***-2*.*7***	***-2*.*2***	***-2*.*4***	***-2*.*5***	***-2*.*9***	
Fmo3	***-2*.*3***		***-2*.*2***	***-3*.*4***		
Gsta1	***-2*.*3***	***-2*.*0***	***-2*.*3***			
Gsta2	***-2*.*1***		***-2*.*3***			
Gsto2		***-2*.*0***			***-2*.*1***	
Sult1e1				3.3		4.7
**VISCERAL ADIPOSE TISSUE**						
**Gene**	**PCB118-10**	**PCB118-100**	**PCB153-10**	**PCB153-100**	**PCB MIX-10**	**PCB MIX-100**
Cyp1a1		5.7				
Cyp11a1						2.5
Fmo3					3.0	
Gpx3	2.7		2.3		2.9	
**MUSCLES**						
**Gene**	**PCB118-10**	**PCB118-100**	**PCB153-10**	**PCB153-100**	**PCB MIX-10**	**PCB MIX-100**
Cyp1a1		15.8				2.6
Gsta2				***-2*.*1***		
Gsto2			***-3*.*1***			
**COLON**						
Cyp1a1						2.9
Cyp11a1	2.6	2.4	2.6	3.2	2.5	2.3

**Table 5:** Genes involved in glucose homeostasis and insulin signaling based on microarray analyses.

**LIVER**						
**Gene**	**PCB118-10**	**PCB118-100**	**PCB153-10**	**PCB153-100**	**PCB MIX-10**	**PCB MIX-100**
Gck	***-4*.*1***	***-4*.*3***	***-3*.*2***	***-3*.*0***		
CnR1	2.9		2.7			
**VISCERAL ADIPOSE TISSUE**						
**Gene**	**PCB118-10**	**PCB118-100**	**PCB153-10**	**PCB153-100**	**PCB MIX-10**	**PCB MIX-100**
Pygb		***-2*.*8***	***-2*.*2***	***-2*.*4***	***-2*.*6***	***-4*.*9***
Decr1	**-2.1**	***-2*.*4***	***-2*.*0***		***-2*.*2***	***-2*.*0***
Foxo3		4.9				
**MUSCLES**						
**Gene**	**PCB118-10**	**PCB118-100**	**PCB153-10**	**PCB153-100**	**PCB MIX-10**	**PCB MIX-100**
Gck	***-2*.*5***	***-2*.*2***			***-2***	***-2***
Foxo3	2.4			3.5	2.4	2.5
Pfkfb3	2.6	2.9	2.1	2.8		
CnR1	2.2		3.2	3.2	2.9	

**Table 6:** Genes involved in lipid homeostasis based on microarray analyses.

**LIVER**						
**Gene**	**PCB118-10**	**PCB118-100**	**PCB153-10**	**PCB153-100**	**PCB MIX-10**	**PCB MIX-100**
Lpin 1	***-3*.*0***	***-2*.*4***	***-2*.*0***	***-2*.*1***		
Pnliprp1		2.9		3.9		4.5
Mogat2	2.3	5.1		3.1	2.4	4.1
Alox5	2.2	2.5		2.4		
Pde8b					2.9	2.8
**VISCERAL ADIPOSE TISSUE**						
**Gene**	**PCB118-10**	**PCB118-100**	**PCB153-10**	**PCB153-100**	**PCB MIX-10**	**PCB MIX-100**
Fasn	***-6*.*5***	***-6*.*6***	***-5*.*2***	***-5*.*7***	***-6*.*8***	
Agpat2	***-2*.*1***	***-2*.*0***				
Gpd1	***-2*.*7***	***-2*.*6***				
Pla2g1b						***-4*.*0***
Pde8b	3.2				5.6	
**MUSCLES**						
**Gene**	**PCB118-10**	**PCB118-100**	**PCB153-10**	**PCB153-100**	**PCB MIX-10**	**PCB MIX-100**
Lipc	***-2*.*3***			***-2*.*8***		2.6
Pla2g1b	***-2*.*8***			***-4*.*1***	***-3*.*4***	
Lpin 2	2.5		2.8	3.0	2.5	2.5
Rgs2	2.6	2.5	2.5	2.1		
Pde8b			2.6			

**Table 7:** Genes involved in inflammation and the immune system based on microarray analyses.

**LIVER**						
**Gene**	**PCB118-10**	**PCB118-100**	**PCB153-10**	**PCB153-100**	**PCB MIX-10**	**PCB MIX-100**
Gata 3	3.8	4.1	2.4	3.1		
PU1	***-2*.*5***			***-2*.*1***		
CD28			***-2*.*0***	***-2*.*0***		
IL1β	2.3					
IL5					***-2*.*5***	***-3*.*4***
Ifng	2.3					***-2*.*8***
Smpd3		2.3		3.8		2.7
**VISCERAL ADIPOSE TISSUE**						
**Gene**	**PCB118-10**	**PCB118-100**	**PCB153-10**	**PCB153-100**	**PCB MIX-10**	**PCB MIX-100**
Serpine 1					2.7	2.8
Slc25a1	***-3*.*7***	***-3*.*1***	***-2*.*9***	***-2*.*7***	***-3*.*8***	
Tank				3.6		
Gata 3						***-18*.*1***
Srgn	3.2		2.7		4.7	2.4
Elk4					2.8	4.7
CD28		***-2*.*0***				
IL5		***-2*.*9***				
**MUSCLES**						
**Gene**	**PCB118-10**	**PCB118-100**	**PCB153-10**	**PCB153-100**	**PCB MIX-10**	**PCB MIX-100**
Serpine 1						2.1
Tank	11.1	7.8	11.7	7.8	12.2	6.6
PU1			***-3*.*9***	***-4*.*8***	***-3*.*2***	
Elk4		3.5		2.9		
IL1β				***-2*.*0***		
IL2	***-2*.*1***		***-2*.*2***	***-2*.*5***		***-3*.*7***
IL5				***-2*.*5***	***-2*.*7***	
IL6		2.9		2.6		
Ifng	***-2*.*4***			***-3*.*5***		
**COLON**						
Tank	4.1	4.0	5.2	7.9	3.7	4.9
PU1	***-2*.*7***				***-2*.*2***	

Table 4 highlights the selected genes involved in the metabolism of xenobiotics. The largest effects were observed in the liver, especially in cytochrome P450s (Cyp). PCB118 enhanced Cyp1a subfamily expression, while PCB153 induced the expression of the Cyp2b subfamily. Interestingly, PCB118 and PCB153, alone or in combination, reduced glutathione S-transferase (GST) expression. In adipose tissue, we observed an induction of the glutathione peroxidase 3 (Gpx3) at the lowest studied dose.

Table 5 summarizes the selected genes involved in glucose homeostasis and insulin signaling. In the liver, we observed a decrease in glucokinase (Gck) expression after PCB118 or PCB153 exposure. In muscles, the decrease in Gck was mediated by PCB118 only. In adipose tissue, glycogen phosphorylase (Pygb) and 2,4-dienoyl CoA reductase 1 (Decr1) were downregulated. In muscles, the expression of 6-phosphofructo-2-kinase/fructose-2,6-biphosphatase 3 (Pfkfb3) and forkhead box O3 (Foxo3) were enhanced. In the liver and muscles, the expression of cannabinoid receptor 1 (Cnr1) was induced. This effect was more pronounced at the lowest studied dose.

Table 6 summarizes the selected genes involved in lipid homeostasis. In the liver, pancreatic lipase related protein 1 (Pnliprp1) and monoacylglycerol O-acyltransferase 2 (Mogat 2) were induced by PCB118 and/or PCB153 exposure, whereas lipin 1 (Lpin 1) expression was downregulated. In adipose tissue, fatty acid synthase (Fasn) expression was reduced by PCB118 and/or PCB153 exposure. In addition, PCB118 reduced the expression of glycerol-3-phosphate dehydrogenase 1 (Gpd1) and 1-acylglycerol-3-phosphate O-acyltransferase 2 (Agpat2). In the muscles, lipin 2 (Lpin2) and regulator of G-protein signaling 2 (Rgs2) expression was increased, and the expression of phospholipase A2, group IB (Pla2g1b) was decreased.

Table 7 shows the results obtained for genes involved in inflammation and the immune system. In the liver, we observed an induction of sphingomyelin phosphodiesterase 3 (Smpd3) after exposure to PCB118 and/or PCB153. GATA binding protein 3 (Gata3) was also induced only after exposure to PCB118 or PCB153 individually. In adipose tissue, an increase in serpine 1 expression was observed after exposure to a mixture of PCB118 and PCB153.

### Real-time RT-PCR results

The results obtained in the liver, visceral adipose tissue, muscles, and colon are summarized in Tables 8–11, respectively.

**Table 8:** Real-time RT-PCR validation of genes selected within the liver after microarray analyses (*p < 0.05).

	C	PCB118-10	PCB118-100	PCB153-10	PCB153-100	PCB MIX-10	PCB MIX-100
Alox5	1.0 ± 0.3	0.5 ± 0.1*	0.4 ± 0.1*	0.4 ± 0.1*	0.3 ± 0.0*	0.5 ± 0.2*	0.5 ± 0.1*
CD28	1.0 ± 0.2	0.7 ± 0.4	1.0 ± 0.0	0.8 ± 0.1	0.7 ± 0.1	1.2 ± 0.2	1.8 ± 0.2*
Cnr1	1.0 ± 0.1	0.4 ± 0.1*	0.8 ± 0.2	0.7 ± 0.1*	0.3 ± 0.0*	1.0 ± 0.0	1.2 ± 0.3
Cyp1a1	1.0 ± 0.2	0.9 ± 0.1	0.7 ± 0.1	0.6 ± 0.0*	0.3 ± 0.1*	0.8 ± 0.0	1.1 ± 0.1
Cyp1a2	1.0 ± 0.1	11.0 ± 3.6*	5.9 ± 1.6*	2.6 ± 0.3*	2.6 ± 0.5*	3.3 ± 0.6*	0.8 ± 0.5
Cyp2b9	1.0 ± 0.1	2.7 ± 0.0*	1.1 ± 0.1	12.3 ± 3.2*	1.8 ± 0.4*	11.0 ± 1.3*	1.4 ± 0.4
Cyp2c54	1.0 ± 0.2	2.0 ± 0.4*	2.0 ± 0.1*	2.6 ± 0.3*	1.3 ± 0.1	1.9 ± 0.3*	1.0 ± 0.5
Cyp2c70	1.0 ± 0.3	0.3 ± 0.0*	0.4 ± 0.1*	0.4 ± 0.0*	0.4 ± 0.1*	0.5 ± 0.2*	0.4 ± 0.1*
Gck	1.0 ± 0.2	0.8 ± 0.3	0.9 ± 0.2	0.9 ± 0.2	0.7 ± 0.0*	1.9 ± 0.7	1.1 ± 0.3
Glut 4	1.0 ± 0.1	0.8 ± 0.1*	1.0 ± 0.1	0.8 ± 0.1*	0.6 ± 0.1*	1.6 ± 0.1*	1.3 ± 0.1*
Gsta1	1.0 ± 0.1	0.6 ± 0.0*	0.5 ± 0.1*	0.5 ± 0.1*	0.4 ± 0.0*	0.6 ± 0.1*	0.6 ± 0.2*
Gsta2	1.0 ± 0.0	0.4 ± 0.1	0.3 ± 0.0	0.4 ± 0.1	0.3 ± 0.1	0.7 ± 0.1	0.5 ± 0.1
Gsto2	1.0 ± 0.1	1.0 ± 0.5	0.5 ± 0.3*	0.4 ± 0.0*	0.6 ± 0.2*	0.4 ± 0.2*	0.3 ± 0.1*
IL-1B	1.0 ± 0.0	1.2 ± 0.1	1.7 ± 0.0*	1.0 ± 0.1	0.9 ± 0.0	2.5 ± 0.9*	1.7 ± 0.1*
Infg	1.0 ± 0.5	0.3 ± 0.1*	0.9 ± 0.1	0.8 ± 0.3	1.1 ± 0.2	1.0 ± 0.3	0.7 ± 0.3
Lipc	1.0 ± 0.2	0.8 ± 0.2	1.7 ± 0.4*	0.8 ± 0.2	0.9 ± 0.2	0.9 ± 0.1	1.1 ± 0.5
Lpin 1	1.0 ± 0.1	0.3 ± 0.1*	0.3 ± 0.0*	0.3 ± 0.1*	0.4 ± 0.1*	0.4 ± 0.1*	0.6 ± 0.0*
Mogat2	1.0 ± 0.1	0.9 ± 0.2	1.4 ± 0.1*	0.8 ± 0.1	1.0 ± 0.2	0.7 ± 0.1	1.1 ± 0.2
Pde8b	1.0 ± 0.1	0.9 ± 0.2	0.9 ± 0.2	1.1 ± 0.2	0.8 ± 0.1	0.9 ± 0.1	0.9 ± 0.2
Pnliprp1	1.0 ± 0.3	0.5 ± 0.2*	0.9 ± 0.2	0.7 ± 0.0*	0.6 ± 0.2*	1.2 ± 0.5	1.3 ± 0.3
Smpd3	1.0 ± 0.1	0.6 ± 0.1*	0.6 ± 0.0*	1.1 ± 0.2	0.6 ± 0.1*	1.1 ± 0.2	0.8 ± 0.1
Sult1e1	1.0 ± 0.3	0.9 ± 0.0	0.8 ± 0.1	2.8 ± 0.3*	3.0 ± 1.9*	2.6 ± 0.5*	1.1 ± 0.3

**Table 9:** Real-time RT-PCR validation of genes selected within the visceral adipose tissue after microarray analyses (*p < 0.05).

	C	PCB118-10	PCB118-100	PCB153-10	PCB153-100	PCB MIX-10	PCB MIX-100
Agpat2	1.0 ± 0.1	0.6 ± 0.0*	0.4 ± 0.1*	0.5 ± 0.2*	0.5 ± 0.0*	0.5 ± 0.1*	0.3 ± 0.1*
CD28	1.0 ± 0.0	0.7 ± 0.4	0.5 ± 0.1*	0.4 ± 0.1*	0.5 ± 0.2*	2.2 ± 0.3*	1.3 ± 0.1
Cyp1a1	1.0 ± 0.5	0.7 ± 0.2	1.0 ± 0.7	0.8 ± 0.1	0.8 ± 0.2	1.4 ± 0.2	0.9 ± 0.2
Eno1	1.0 ± 0.5	0.4 ± 0.1*	0.3 ± 0.0*	0.4 ± 0.1*	0.3 ± 0.0*	0.6 ± 0.1*	0.5 ± 0.0*
Fasn	1.0 ± 0.1	3.7 ± 4.1	1.8 ± 1.0*	0.4 ± 0.1*	0.4 ± 0.2*	2.6 ± 0.6*	1.5 ± 0.3*
Foxo3	1.0 ± 0.4	0.8 ± 0.2	0.7 ± 0.0	0.8 ± 0.1	0.7 ± 0.2	0.9 ± 0.2	0.8 ± 0.3
Glut4	1.0 ± 0.3	0.5 ± 0.0*	0.6 ± 0.1*	0.5 ± 0.2*	0.5 ± 0.2*	1.1 ± 0.1	0.7 ± 0.2
Gpx3	1.0 ± 0.1	0.8 ± 0.1	2.2 ± 0.6*	1.2 ± 0.4	1.7 ± 0.8	1.2 ± 0.2	3.8 ± 1.2*
IL-1B	1.0 ± 0.2	0.3 ± 0.0*	0.3 ± 0.1*	0.2 ± 0.1*	0.2 ± 0.1*	1.7 ± 0.8	0.9 ± 0.1
Lpin1	1.0 ± 0.1	0.7 ± 0.1*	0.4 ± 0.0*	1.2 ± 0.5	0.7 ± 0.4	1.3 ± 0.4	0.9 ± 0.2
Pde8b	1.0 ± 0.1	0.9 ± 0.0	0.8 ± 0.1	0.8 ± 0.0	0.7 ± 0.2	1.8 ± 0.4*	1.0 ± 0.2
Pla2g1b	1.0 ± 0.3	1.6 ± 0.4	1.1 ± 0.3	1.1 ± 0.4	1.1 ± 0.4	3.8 ± 1.0*	1.9 ± 0.3
Pygb	1.0 ± 0.1	1.0 ± 0.4	0.8 ± 0.0*	0.9 ± 0.1	0.9 ± 0.3	0.7 ± 0.1*	0.8 ± 0.4
Serpine 1	1.0 ± 0.1	0.7 ± 0.1*	0.9 ± 0.4	1.4 ± 0.1*	0.7 ± 0.3	1.5 ± 0.4	2.0 ± 1.0
Slc25a1	1.0 ± 0.2	0.4 ± 0.0*	0.5 ± 0.2*	0.4 ± 0.1*	0.4 ± 0.1*	0.7 ± 0.2	0.5 ± 0.0*
Tank	1.0 ± 0.1	0.6 ± 0.0*	0.8 ± 0.1	0.9 ± 0.2	0.7 ± 0.2	0.8 ± 0.2	0.7 ± 0.3

**Table 10:** Real-time RT-PCR validation of genes selected within the muscles after microarray analyses (*p < 0.05).

	C	PCB118-10	PCB118-100	PCB153-10	PCB153-100	PCB MIX-10	PCB MIX-100
CD28	1.0 ± 0.9	1.6 ± 2.6	0.9 ± 1.1	0.2 ± 0.0	0.4 ± 0.1	0.4 ± 0.1	0.0 ± 0.0
Cnr1	1.0 ± 0.2	1.3 ± 0.1	1.5 ± 0.0*	3.8 ± 1.8*	20.1 ± 13.7*	13.1 ± 2.1*	1.6 ± 0.2*
Cyp1a1	1.0 ± 0.1	1.7 ± 0.3*	1.7 ± 1.3	1.4 ± 0.5	1.6 ± 0.8	1.1 ± 0.3	1.2 ± 0.1
Foxo3	1.0 ± 0.4	1.7 ± 0.3	1.4 ± 0.2	1.1 ± 0.1	1.5 ± 0.2	1.3 ± 0.1	1.0 ± 0.2
Gck	1.0 ± 0.3	1.1 ± 0.0	3.1 ± 3.3	2.1 ± 0.9	1.9 ± 1.2	2.0 ± 1.2	1.5 ± 0.1
Glut4	1.0 ± 0.3	1.1 ± 0.3	1.2 ± 0.1	1.3 ± 0.1	1.2 ± 0.4	1.0 ± 0.2	1.2 ± 0.1
Gsta2	1.0 ± 0.1	1.0 ± 0.1	1.9 ± 0.9	3.3 ± 2.2	6.4 ± 1.7*	6.7 ± 2.5*	1.0 ± 0.2
Gyk	1.0 ± 0.8	0.6 ± 0.0	0.8 ± 0.2	1.0 ± 0.4	1.3 ± 0.6	0.8 ± 0.2	0.9 ± 0.0
Ifng	1.0 ± 0.2	2.0 ± 0.5	0.5 ± 0.6	0.1 ± 0.1	0.5 ± 0.7	0.3 ± 0.2	0.1 ± 0.1
IL-1beta	1.0 ± 0.2	1.4 ± 0.3	1.5 ± 0.0*	2.4 ± 1.1*	2.5 ± 1.3	3.5 ± 2.4	1.6 ± 0.2*
IL2	1.0 ± 0.2	2.5 ± 2.2	0.9 ± 1.1	0.2 ± 0.1*	1.2 ± 1.4	0.3 ± 0.2*	ND
IL6	1.0 ± 0.8	0.1 ± 0.0*	0.9 ± 1.1	0.1 ± 0.1*	1.0 ± 1.0	0.5 ± 0.2	0.1 ± 0.0
Lipc	1.0 ± 0.3	1.3 ± 0.3	2.2 ± 1.1	1.8 ± 0.8	1.9 ± 1.2	1.4 ± 0.4	1.9 ± 0.4*
Lpin2	1.0 ± 0.0	3.7 ± 1.3*	4.3 ± 3.0*	3.3 ± 0.8*	3.3 ± 0.2*	2.7 ± 0.4*	2.5 ± 1.3*
Pde8b	1.0 ± 0.2	1.4 ± 0.2	1.2 ± 0.3	1.5 ± 0.1*	1.5 ± 0.1*	1.9 ± 0.3*	1.2 ± 0.2
Pfkfb3	1.0 ± 0.1	3.4 ± 1.4*	3.5 ± 0.8*	2.8 ± 0.4*	3.5 ± 1.2*	2.1 ± 0.7*	1.8 ± 0.5*
Pla2g1b	1.0 ± 1.3	0.0 ± 0.0*	0.1 ± 0.0*	0.3 ± 0.4*	0.9 ± 0.8	0.7 ± 0.1	0.0 ± 0.0*
Rgs2	1.0 ± 0.1	1.5 ± 0.1*	2.0 ± 0.2*	3.7 ± 1.2*	18.4 ± 7.2*	11.0 ± 5.9*	1.6 ± 0.2*
Serpine 1	1.0 ± 1.1	0.2 ± 0.0*	0.3 ± 0.2*	0.2 ± 0.1*	0.3 ± 0.1*	0.5 ± 0.1*	0.3 ± 0.1*

**Table 11:** Real-time RT-PCR validation of genes selected within the colon after microarray analyses (*p < 0.05).

	C	PCB118-10	PCB118-100	PCB153-10	PCB153-100	PCB MIX-10	PCB MIX-100
Cyp1a1	1.0 ± 0.1	1.4 ± 0.2*	1.7 ± 0.6	1.4 ± 0.4	2.6 ± 0.6*	1.3 ± 0.2	1.5 ± 0.3
Cyp2c70	1.0 ± 0.2	1.8 ± 0.3*	0.5 ± 0.0*	1.9 ± 0.4*	1.2 ± 0.2	1.4 ± 0.9	1.4 ± 0.2
Glut4	1.0 ± 0.2	0.7 ± 0.1	0.9 ± 0.3	0.8 ± 0.2	0.9 ± 0.2	0.9 ± 0.2	0.9 ± 0.1
IL-1B	1.0 ± 0.2	0.5 ± 0.1*	0.5 ± 0.0*	0.8 ± 0.3	0.8 ± 0.1	0.7 ± 0.2	0.9 ± 0.2
Serpine 1	1.0 ± 0.3	1.4 ± 0.0*	0.5 ± 0.2	1.5 ± 0.3	1.3 ± 0.4	1.6 ± 0.9	1.6 ± 0.7
Tank	1.0 ± 0.1	1.0 ± 0.3	0.4 ± 0.1*	1.2 ± 0.1	1.4 ± 0.1*	1.0 ± 0.2	1.2 ± 0.2

For genes involved in the metabolism of xenobiotics, our results confirmed the hepatic induction of Cyp1a2, Cyp2b9, Cyp2c54 and the repression of Cyp2c70. No significant modification was observed in the hepatic expression of Cyp1a1; however, this isoform is mainly expressed in extrahepatic tissues. In the colon, Cyp1a1 and Cyp2c70 expression was slightly increased. We confirmed the downregulation of Gsta1, Gsta2, and Gsto2 expression in the liver. Surprisingly, we observed an increase in Gsta2 expression in muscles. Moreover, PCB153 alone or in combination with PCB118 enhanced Sult1e1 expression.

Within the changes in gene expression for glucose homeostasis and insulin signaling, the data for Gck are conflicting. The microarray analysis showed a decrease in Gck expression in the liver and muscles. However, qRT-PCR analysis showed only a slight variation in the liver and an increase in the muscle. For CnR1, our data confirmed the induction of its expression in muscles, and PCB153 exerted a stronger effect when compared with PCB118. CnR1 hepatic expression was reduced, except after exposure to the mixture of PCBs. We confirmed the decrease in Foxo3 and Pfkfb3 in the muscles. Moreover, we used qRT-PCR to verify changes in Glut4 expression because previous studied showed its decrease in 3T3 L1 after TCDD exposure [4]. After PCB exposure, Glut4 expression showed minor changes in the liver, muscle and colon; however, Glut4 was clearly downregulated in adipose tissue. Pygb was downregulated in this tissue to a lesser extent than previously observed using microarray analyses.

For genes involved in lipid homeostasis, qRT-PCR analysis confirmed a decrease in lpin 1 expression in liver and adipose tissue and an increase in muscular lpin2 expression. Agpat2 expression was decreased in adipose tissue after exposure to PCB. The induction of Rsg2 and a decrease in Pla2g1b in the muscles was confirmed. Fasn expression was decreased in adipose tissue after exposure to PCB153; however, exposure to PCB118 alone or in combination caused an increase in Fasn expression. For Gyk, the qRT-PCR analysis did not confirm an increase in its expression in muscles after exposure to a mixture of PCB118 and PCB153. Exposure to PCB118 produced a decrease in Gyk expression. Similarly, there were some discrepancies with the results obtained for Alox5, Mogat2, Pnliprp1 and Pde8b. qRT-PCR analysis showed a marked decrease in Alox5 hepatic expression after PCB118 or PCB153 exposure; however, the microarray data showed an induction of Alox5. For Mogat2, the microarray data showed an increase in hepatic expression; however, we observed only minor changes in expression using qRT-PCR.

Analysis of microarray or qRT-PCR results did not show significant changes in genes involved in inflammation or the immune system, such as pro-inflammatory cytokines and chemokines. A mild increase in serpine 1 was found in adipose tissue after exposure to the mixture of PCB118 and PCB153. Minor changes in hepatic Smpd3 were not confirmed. Tank expression in the colon remained stable, and data obtained from muscles suggest that Tank is poorly expressed (Cp ≥ 35 cycles).

## Discussion

Except for a significant increase in triglyceridemia after exposure to PCB118 and/or PCB153, we did not observe any changes in the other selected biochemical parameters. The greatest induction of triglyceridemia was found in groups exposed to the lowest doses of PCB118 or PCB153. Hypertriglyceridemia observed after PCB exposure could be explained by insulin resistance. Unfortunatly, we did not get enough material to determine insulinemia, and we cannot calculate HOMA-IR in order to evaluate peripheral insulin resistance.

For genes related to xenobiotic metabolism, we observed an induction of Cyp1a2 in the liver and Cyp1a1 in the colon after exposure to PCB118. The induction of the Cyp1 family by PCB-DL has been previously described. Our data demonstrated that non-DL PCBs, such as PCB153, can also induce Cyp1a expression, albeit to a lesser extent. Interestingly, the increase in Cyp1a expression was associated with a decrease in Gst expression. Cyp1a catalyzes the bioactivation of various environmental procancerogens (such as PAHs and arylamines present in our diet) into electrophilic metabolites [12]. Typically, these reactive metabolites are further detoxified by Gst [13]. Therefore, our data suggest that PCB exposure increases bioactivation reactions while reducing the detoxification process, which leads to an increased susceptibility to procarcinogens. This phenomenon could explain the carcinogenic effects of PCB. Moreover, electrophile metabolites can induce inflammatory responses [14], and the resulting hepatic inflammation could be partly responsible for the metabolic side effects of PCB. The hepatic induction of Cyp2b expression after PCB153 exposure has been previously described in rats [15]. The human ortholog CYP2B6 is involved in the hepatic degradation of certain drugs [16], and exposure to non-DL PCBs could modify the pharmacokinetic of such drugs. In the liver, we observed an induction of estrogen sulfotransferase (Sult1e1) after exposure to PCB153 either alone or in combination with PCB118. Sult1e1 expression is regulated by CAR in mice [17], and it was recently suggested that Sult1e1 functions as a negative regulator of adipogenesis [18].

For genes involved in glucose homeostasis, insulin signaling, lipid homeostasis and inflammation, we have focused on CnR1, lipin 1 and 2, Glut4, Agpat2, Slc25a1, Fasn and Foxo 3.

Recent studies have shown that the endocannabinoid system has significant effects on energy balance and metabolism through the central control of appetite and peripheral metabolism [19]. PCB exposure slightly modified the hepatic expression of CnR1; however, PCB153 alone or with PCB118 induced a potent increase in CnR1 expression in muscles. Interestingly, in humans, CnR1 participates in the negative crosstalk between fat and keletal muscle cells, and recent data suggest it may play a role in the development of insulin resistance [20]. Therefore, CnR1 may be partly involved in some of metabolic side effects of PCB.

Our results showed that exposure to PCB118 and/or PCB153 reduced the expression of Lipin 1 in the liver and, to a lesser extent, in adipose tissue. Studies of lipin 1–deficient mice and cells have shown that lipin 1 is required for adipocyte differentiation. Lipin 1–deficient cells and tissues failed to induce expression of two key transcription factors of adipocyte differentiation, PPARγ and C/EBPα. Instead, these cells expressed high levels of preadipocyte factor-1, an inhibitor of adipogenesis [21]. A strong negative correlation between lipin 1 mRNA levels in adipose tissue and glucose levels, insulin levels, and insulin resistance has been described in mice and humans [22]. In humans, adipose Lipin 1 gene expression was strongly associated with both basal and insulin-mediated subcutaneous adipocyte glucose transport and mRNA levels of Glut4 [23]. Interestingly, we also observed a decrease in Glut4 expression in adipose tissue after exposure to PCB118 and/or PCB153. In addition, we showed a less pronounced downregulation of Glut4 expression in other tissues. The insulin-regulated glucose transporter Glut4 is mainly expressed in insulin-responsive tissues, i.e., white and brown adipose tissue and heart and skeletal muscles. Glut4 mediates glucose uptake in response to acute insulin stimulation [24]. Insulin-regulated glucose uptake in skeletal muscle and adipose tissue is dependent on the redistribution of Glut4 from intracellular storage sites to the plasma membrane [25]. Impaired insulin stimulation of glucose uptake in adipose tissue and skeletal muscle is one of the earliest defects detected in insulin-resistant states. Insulin-resistance in type 2 diabetes, obesity, and aging is associated with a marked reduction in the intracellular pool of Glut4 protein in adipose cells, which in turn impairs insulin stimulation of glucose transport [26].

Exposure to PCB118, and/or PCB153 enhanced the expression of Lipin 2 in muscles. Lipin-2 is prominently expressed in the liver, and its hepatic expression is induced in mice by fasting and diet-induced obesity [27]. Lipin 2 induction in the liver is associated to hepatic insulin resistance [28]. However, little is known concerning the role of lipin 2 in muscles.

Agpat2 expression was downregulated in adipose tissue after exposure to PCB118 and/or PCB153. AGPATs catalyze the conversion of lysophosphatidic acid to phosphatidic acid and play a critical role in the biosynthesis of glycerophospholipids and triglycerides from glycerol-3-phosphate [29]. A role for Agpat2 in triglyceride synthesis and adipocyte biology has emerged since the discovery of mutations in *AGPAT2* caused an autosomal recessive form of congenital generalized lipodystrophy (CGL) type 1. Patients with CGL are born with a near complete absence of adipose tissue and are prone to develop metabolic complications associated with insulin resistance, such as impaired glucose tolerance, diabetes, hypertriglyceridemia, and hepatic steatosis early in life [30]. Similarly, Agpat2^−/−^ mice develop severe lipodystrophy affecting both white and brown adipose tissues, severe insulin resistance, diabetes, and hepatic steatosis [31]. The results obtained in 3T3-L1 preadipocytes after knockdown or overexpression of Agpat2 suggested that Agpat2 regulates adipogenesis through the modulation of the lipoma and by altering the normal activation of phosphatidylinositol 3-kinase (PI3K)/Akt and PPARγ pathways in the early stages of adipogenesis [32]. Therefore, Agpat2 may be partly involved in the metabolic side effects of PCB.

We also observed a decrease in Slc25a1 expression in adipose tissue after PCB exposure. Slc25a1 is a member of the mitochondrial carrier subfamily of solute carrier proteins and is an essential component of the shuttle system that transports acetyl-CoA from the mitochondria to the cytosol for lipogenesis. Slc25a1 plays an important role in glucose-stimulated insulin secretion [33]. Slc25a1 is regulated by SREBP-1, as well as PPARα and PPARγ in hepatocytes and adipocytes, respectively [34]. Moreover, the activation of AhR by TCDD suppresses PPARγ_1_ expression and subsequent adipocyte differentiation in C3H10T1/2 cells [35]. Therefore, PCB-DL exposure may downregulate PPARγ in adipose tissue, leading to a decrease in Slc25a1.

Fasn is a key enzyme required for the de novo synthesis of fatty acids. Fasn was induced in adipose tissue after exposure to PCB118 alone or in combination with PCB153. In murine adipose tissue, large adipocytes have a higher expression of Fasn and are more insulin-resistant than small adipocytes [36]. In humans, increased FASN gene expression in adipose tissue is linked to visceral fat accumulation, impaired insulin sensitivity, increased circulating fasting insulin, IL-6, leptin and RBP4 [37]. Therefore, Fasn may be partly involved in the metabolic effects of PCB.

In muscles, we observed an induction of Foxo3 expression. Foxo family member proteins are highly conserved transcription factors with important roles in cellular homeostasis, especially skeletal muscle. Foxo1 and Foxo3 are key players in muscle energy homeostasis through the control of glycolytic and lipolytic flux and mitochondrial metabolism. Their exacerbated activation occurs in several diseases and results in atrophy, mitochondrial dysfunction, and a detrimental shift in the muscle phenotype [38–39]. Therefore, Foxo3 may be partly involved in PCB-induced metabolic diseases. However, additional studies are needed to elucidate the precise role of these proteins in mitochondrial homeostasis.

In our environment we are exposed to mixtures of PCBs, therefore we have evaluated the effect of an exposition to an equimolar mixture of PCB118 and PCB153. We failed to observe a marked differences between the effects of the mixture and the effecs of PCB118 or PCB153 alone, even with genes involved in xenobiotic metabolism, where we have observed the strongest modifications of gene expression.

In summary, our results showed that a short-term exposure to PCB118, PCB153, or a mixture of PCB118 and PCB153 enhanced circulating triglyceride levels without affecting glycemia. Interestingly, the strongest effects were produced by the lowest studied dose of PCB. This phenomenon has already been described for numerous endocrine disruption processes. Among the studied tissues, we did not observe changes in inflammation-related genes, such as cytokines or chemokines. The main transcriptional effects were observed in visceral adipose tissue and the liver. We found a downregulation of lipin1 and glut4 expression in these two target organs. In adipose tissue, we also showed a downregulation of Agpat2, Slc25a1, and Fasn. These genes are involved in lipid metabolism and are associated with insulin resistance. In muscles, we observed an induction of CnR1 and Foxo3 expression. The induction of Foxo3 suggests that PCB may induce mitochondrial dysfunction in muscles. The metabolic side-effects of PCB may be due to the modulation of CnR1 in the hypothalamus which controls appetite and regulates AMPK activity [40]. Moreover, we are exposed to various exogenous compounds, including procancerogens such as PAH and arylamines. Their bioactivation is catalyzed by Cyp1a which induces inflammatory responses [14] and may enhance the metabolic effects of PCB. Indeed, type 2 diabetes is linked to the low grade inflammation of visceral adipose tissue. While our results suggest that adipocytes are the main target of the metabolic disorders induced by PCB, further studies are required to fully elucidate these mechanisms, e.g., the link between lipin 1 and Glut4 expression and the redistribution of Glut4 from intracellular storage sites to the plasma membrane. Moreover, it would be of interest to further characterize the synergistic effects and epigenetic regulation of a combined PCB118 and PCB153 exposure.

## International Gene Abbreviations Used in Tables

*Agpat2*: 1-acylglycerol-3-phosphate O-acyltransferase 2. *Alox5*: arachidonate 5-lipoxygenase. *CD28*: CD28 antigen. *CnR1*: cannabinoid receptor 1. *Cyp11a1*: Cytochrome P450 11a1. *Cyp1a1*: cytochrome P450 1a1. *Cyp1a2*: cytochrome P450 1a2. *Cyp2b10*: cytochrome P450 2b10. *Cyp2b9*: cytochrome P450 2b9. *Cyp2c54*: cytochrome P450 2c54. *Cyp2c70*: cytochrome P450 2c70. *Decr1*: 2,4-dienoyl CoA reductase 1. *Elk4*: ETS-domain protein (SRF accessory protein 1). *Eno1*: enolase 1. *Fasn*: fatty acid synthase. *Fmo3*: flavin containing monooxygenase 3. *Foxo3*: forkhead box O3. *Gata3*: GATA binding protein 3. *Gck*: glucokinase. *Glut4*: solute carrier family 2 (facilitated glucose transporter), member 4. *Gpd1*: glycerol-3-phosphate dehydrogenase 1. *Gpx3*: glutathione peroxidase 3. *Gsta1*: glutathione S-transferase alpha 1. *Gsta2*: glutathione S-transferase alpha 2. *Gsto2*: glutathione S-transferase omega 2. *Gyk*: glycerol kinase. *Ifng*: interferon gamma. *IL1β*: interleukin 1 beta. *IL2*: interleukin 2. *IL5*: interleukin 5. *IL6*: interleukin 6. *Lipc*: lipase. *Lpin1*: lipin 1. *Lpin2*: lipin 2. *Mogat2*: monoacylglycerol O-acyltransferase 2. *Pde8b*: phosphodiesterase 8B. *Pfkfb3*: 6-phosphofructo-2-kinase/fructose-2,6-biphosphatase 3. *Pla2g1b*: phospholipase A2, group IB. *Pnliprp1*: pancreatic lipase related protein 1. *PU1*: Spi-1 proto-oncogene b. *Pygb*: brain glycogen phosphorylase. *Rgs2*: regulator of G-protein signaling 2. *Slc25a1*: solute carrier family 25 (mitochondrial carrier; citrate transporter), member 1. *Smpd3*: sphingomyelin phosphodiesterase 3. *Srgn*: serglycin. *Sult1e1*: sulfotransferase family 1E. *Tank*: TRAF family member-associated NFκB activator.

## Supporting Information

S1 File Genes highlighted after DNA cheap analysis. Abbrevations: Liver 118–10: results obtained in liver after treatment with PCB118 10 μmol/Kg bw. Liver 118–100: results obtained in liver after treatment with PCB118 100 μmol/Kg bw. Liver 153–10: results obtained in liver after treatment with PCB153 10 μmol/Kg bw. Liver 153–100: results obtained in liver after treatment with PCB153 100 μmol/Kg bw. Liver MIX-10: results obtained in liver after treatment with an equimolar mixture of PCB118 and PCB153 10 μmol/Kg bw. Liver MIX-100: results obtained in liver after treatment with an equimolar mixture of PCB118 and PCB153 100 μmol/Kg bw. Colon 118–10: results obtained in colon after treatment with PCB118 10 μmol/Kg bw. Colon 118–100: results obtained in colon after treatment with PCB118 100 μmol/Kg bw. Colon 153–10: results obtained in colon after treatment with PCB153 10 μmol/Kg bw. Colon 153–100: results obtained in colon after treatment with PCB153 100 μmol/Kg bw. Colon MIX-10: results obtained in colon after treatment with an equimolar mixture of PCB118 and PCB153 10 μmol/Kg bw. Colon MIX-100: results obtained in colon after treatment with an equimolar mixture of PCB118 and PCB153 100 μmol/Kg bw. Adipose 118–10: results obtained in visceral adipose tissue after treatment with PCB118 10 μmol/Kg bw. Adipose 118–100: results obtained in visceral adipose tissue after treatment with PCB118 100 μmol/Kg bw. Adipose 153–10: results obtained in visceral adipose tissue after treatment with PCB153 10 μmol/Kg bw. Adipose 153–100: results obtained in visceral adipose tissue after treatment with PCB153 100 μmol/Kg bw. Adipose MIX-10: results obtained in visceral adipose tissue after treatment with an equimolar mixture of PCB118 and PCB153 10 μmol/Kg bw. Adipose MIX-100: results obtained in visceral adipose tissue after treatment with an equimolar mixture of PCB118 and PCB153 100 μmol/Kg bw. Muscle 118–10: results obtained in muscle after treatment with PCB118 10 μmol/Kg bw. Muscle 118–100: results obtained in muscle after treatment with PCB118 100 μmol/Kg bw. Muscle 153–10: results obtained in muscle after treatment with PCB153 10 μmol/Kg bw. Muscle 153–100: results obtained in muscle after treatment with PCB153 100 μmol/Kg bw. Muscle MIX-10: results obtained in muscle after treatment with an equimolar mixture of PCB118 and PCB153 10 μmol/Kg bw. Muscle MIX-100: results obtained in muscle after treatment with an equimolar mixture of PCB118 and PCB153 100 μmol/Kg bw.
(XLSX)
